# Nursing Students’ Attitudes towards Immigrants’ Social Rights

**DOI:** 10.3390/ijerph17238875

**Published:** 2020-11-29

**Authors:** María Angustias Sánchez-Ojeda, Silvia Navarro-Prado, Adelina Martín-Salvador, Trinidad Luque-Vara, Elisabet Fernández-Gómez, Fernando Jesús Plaza del Pino

**Affiliations:** 1 Department of Nursing, Faculty of Health Sciences, University of Granada, 52017 Melilla, Spain; maso@ugr.es (M.A.S.-O.); ademartin@ugr.es (A.M.-S.); triluva@ugr.es (T.L.-V.); elisabetfdez@ugr.es (E.F.-G.); 2 Department of Nursing, Faculty of Health Sciences, University of Almería, 04120 Almería, Spain; ferplaza@ual.es

**Keywords:** students, nursing, attitudes, rights, publicly funded healthcare

## Abstract

The migrant population has increased in recent years and, as a result, so has cultural diversity. Universities are incorporating specific modules addressing cultural diversity. However, the native population has negative attitudes towards immigrants, as they believe that immigrants receive more social benefits and abuse healthcare services. Nurses may have these attitudes too, which may affect the way they treat patients. The objective of this study was to determine nursing students’ attitudes towards the rights of the migrant population. This is a descriptive ex post facto study using a cross-sectional design, with 821 nursing students in Melilla, Ceuta, and Almeria, Spain. An anonymous questionnaire was used for data collection. Students recognize the same rights for both the immigrant and native populations. More than 80% of the sample upholds the right of undocumented immigrants and their families to access publicly funded healthcare. Attitudes were more positive among students with a Berber background and first-year students. Students approved of the right of immigrants and their families to healthcare and education. The students’ negative attitudes towards the social rights of immigrants need to be addressed with intercultural training to reduce their prejudices as future professionals in a multicultural society.

## 1. Introduction

The global number of international migrants has reached 272 million: 3.50% of the world’s population and an increase of 52 million people since 2010 [1]. Migration flows lead to a gradual increase in cultural and religious diversity in the recipient countries [2]. Various studies have shown how cultural and linguistic diversity compromises the quality of care offered by the healthcare services [3,4,5] and hinder patients’ access to the healthcare system [6], to the point that foreign patients are considered as complicated patients [7] and problematic patients [8].

The ethnocultural profiles of nurses and hospital populations in Spain have been largely homogeneous until recently, with the majority being Spanish citizens of Catholic tradition [5]. However, migration has caused cultural diversity to increase progressively in the Spanish healthcare services. Since the year 2000, Spain has experienced one of the largest increases in immigration in the European Union, with the number of foreign residents rising from 923,879 to 5,650,968 [9].

There is a growing concern about the need to improve the intercultural training of nurses in order to respond to this new reality [4,10,11,12]. Nursing studies in Spain have introduced subjects addressing cultural diversity [13] with the aim of developing intercultural skills in nursing students [14] and improve clinical practice [15].

The coasts of southern Spain, along with the Spanish cities of Ceuta and Melilla in North Africa, are the main entry points for Africans wanting to reach Europe [13,16], using Spain as a transit country [17]. It is in these regions where we have developed this research.

Ceuta and Melilla are two Spanish cities in North Africa where four cultures coexist: Christians of European origin and Christian religion; Hebrews; Romani; and Berbers of Muslim religion and Tamazight language, with Europeans and Berbers being the most numerous groups [13]. In Ceuta, Muslims make up 44% of the population (38% indigenous and 6% immigrants, mainly Moroccans). In Melilla, Muslims make up 52% of the population (38% native and 14% immigrant, mainly Moroccans) [18], not including the thousands of Moroccans who cross the borders of both cities every day, as citizens of both countries are permitted to traverse the Spanish-Moroccan border freely. This situation means that the healthcare system in Ceuta and Melilla is overburdened by their Moroccan neighbors, especially regarding emergencies and childbirth care [19].

Almeria, on the southern coast of Spain, is the province of Andalusia with the largest number of foreign residents (20.28%) [20], as a result of it being the “Southern Border” of Europe and having intensive agriculture with more than 31,000 hectares of greenhouses [21] with a high demand for manpower.

Although the existing data disprove these beliefs, recent studies confirm that there is a growing perception among the native population that foreign residents abuse healthcare services and have greater social protection [22,23,24].

Nurses, like the rest of the population, hold prejudices that affect their care practice and the way they deal with their migrant patients [25,26,27], leading to health inequalities [28]. A previous study [29] exploring nurses’ attitudes towards immigrant patients revealed that 15.8% of the sample held clearly negative attitudes towards immigrants, with Moroccan patients scoring the lowest. Due to its potential implications for clinical practice, we considered it to be of paramount importance to assess how the social rights of the migrant population are regarded by students at the nursing faculties of Ceuta, Melilla, and Almeria, where cultural diversity is very much present.

The objective of this study was to determine nursing students’ attitudes towards the rights of the migrant population.

## 2. Materials and Methods

### 2.1. Study Design

This is a descriptive ex post facto study using a cross-sectional design. The following study variables, based on the socio-demographic data collected alongside the instrument, were considered:

- Socio-demographic variables: sex, cultural background, academic year, and faculty.
- Dependent variables: the nursing students’ attitudes towards the social rights of immigrants.

### 2.2. Participants

The present study was conducted in the Faculty of Health Sciences of the University of Almeria and in two Faculties of Health Sciences of the University of Granada (at the Melilla and Ceuta campuses) during the first semester of the 2019–2020 academic year. Participation was voluntary and anonymous. The participants were 821 nursing students: 282 from Melilla, 239 from Ceuta, and 300 from Almeria. Of the sample, 78.70% were female, 89.30% reported that they were of European origin, 7.90% reported that they were of Berber origin, and the rest reported having other cultural backgrounds. In terms of academic year, 31.40% of the sample were first-year students, 24.50% were second-year students, 23.80% were third-year students, and 20.30% were fourth-year students.

### 2.3. Instrument

The Scale of Attitudes towards Immigration in Nursing (EAIE, by its Spanish acronym), by Antonín and Tomás-Sábado (2004) [30], as modified by Plaza del Pino et al. (2007) [29], was used. This scale assesses positive and negative attitudes towards immigration, with items expressing typical ways of thinking or feeling. Plaza del Pino et al. [29] organized the EAIE in four dimensions or subscales: “immigration and culture”, “immigration and social rights”, “immigration and prejudice”, and “immigration and integration”. A 4-point Likert scale was used, ranging from “Strongly disagree” (SD) to “Strongly agree” (SA). Higher scores indicate poorer attitudes. The questionnaire consists of 39 items.

In this study, the subscale “immigration and social rights” was used. It is made up of 12 items: 5 positive attitudes and 7 negative attitudes.

### 2.4. Procedure

Intentional non-probabilistic sampling was used to obtain the sample. Authorization to recruit students was obtained from the different faculties. Data collection took place during the first semester of the 2019–2020 academic year. Students were recruited from the compulsory modules with the highest levels of student attendance to ensure maximum student participation. A member of the research group explained the research objectives to the students and invited them and their instructors to participate. The questionnaire was completely anonymous and confidential.

### 2.5. Data Analysis

Firstly, the reliability of the scale was analyzed using Cronbach’s alpha, which yielded a value of 0.959, indicating a high level of reliability. For the subscale “immigration and social rights”, a Cronbach’s alpha of 0.864 was obtained, which was also very high.

Subsequently, a descriptive analysis was performed using percentages. For inferential statistics, a non-parametric test was conducted due to the non-normal distribution of the data. Differences in scale values by culture and sex were explored using the Mann-Whitney *U*-test. Differences in scale values by academic year and faculty were explored using the Kruskall-Wallis test. The statistical significance threshold was set at *p* ≤ 0.05. Data were treated with the Statistical Package for the Social Sciences (SPSS) program, version 25, (IBM, New York, NY, USA, for, Windows).

### 2.6. Ethical Considerations

The present study was conducted in compliance with the ethical principles set out in the Declaration of Helsinki. The research protocol was approved by the Research Ethics Committees of the nursing departments of Melilla, Ceuta, and Almeria, Spain (52/2019). In addition, students participated voluntarily having signed an informed consent form. The confidentiality of the data and the anonymity of the participants were preserved at all times.

## 3. Results

In order to meet the study objectives, the nursing students’ attitudes towards the social rights of immigrants were analyzed.

As shown in Table 1, the percentages for the responses obtained for each of the selected items were calculated.

As shown in Table 1, the students surveyed are in favor of incorporating immigrants into society as citizens with full rights and equal working conditions and recognize that the host society has to make a greater effort to improve the quality of life of immigrants. The undocumented immigrants’ right to access publicly funded healthcare and education is approved of by 80% of the students.

The total scores of the analyzed items were calculated, as well as the mean, minimum, and maximum scores, and the 50th and 75th percentiles (Table 2).

Of the sample, 43.97% was below the 50th percentile, with low scores, while 24.48% was above the 75th percentile.

Inferential analyses of the items were conducted according to the study variables: sex, cultural background, academic year, and faculty. The following tables include statistically significant results only.

- ***Variable: sex.*** No statistically significant differences based on the students’ sex were found with respect to the social rights of immigrants.
- ***Variable: cultural background***. Only the European and Berber cultural backgrounds were taken into account, as they were the most numerous. Significant differences were found in the items showing negative attitudes, with students of Berber origin having a better attitude than those of European origin (Table 3).
- ***Variable: academic year***. Statistically significant differences were found only in two items that showed positive attitudes: “Immigrants should enjoy the same working conditions as nationals do” and “All the people living in the same country, regardless of their origins or ethnicity, should have equal rights and obligations”. Third-year and fourth-year students had more prejudice regarding the first item, while second-year and fourth-year students had more prejudice regarding the second item. First-year students displayed a better attitude with respect to both items (Table 4).
- ***Variable: faculty.*** Statistically significant differences were observed in three positive items: “It is desirable that immigrants be incorporated into our society as citizens with full rights”, “Immigrants should enjoy the same working conditions as nationals do”, and “All the people living in the same country, regardless of their origins or ethnicity, should have equal rights and obligations”, with students in Almeria displaying the best attitude. Statistically significant differences were observed in all the negative items, with students in Melilla and Ceuta having the poorest attitude (Table 5).

## 4. Discussion

The purpose of this study was to analyze the nursing students’ attitudes towards the social rights of the migrant population in three Spanish faculties, two located in North Africa (Ceuta and Melilla) and another one located on the Mediterranean coast (Almeria).

Overall, the results indicate that a large majority of students recognize that immigrants have the same rights as the native population, including the right to equal working conditions, which is in consonance with studies such as that of Plaza del Pino and Martínez [31]. In terms of access to public healthcare, Spanish students exhibit a higher percentage of approval than students from Portugal [32] and nurses [31], despite Spain having a larger foreign population in percentage terms (12.9%) than Portugal (8.6%) [32]. This is an important fact, since Spain recognizes undocumented immigrants’ right to publicly funded healthcare [33]. As for the right to education for the children of undocumented immigrants, percentages are lower. Even though it is still a majority of students who uphold this right, this percentage is lower than in other studies [31,32].

No significant differences by sex were found, coinciding with some other studies [32,34,35] and contrary to other studies [13,36].

With respect to the cultural background, most Berber students had a better attitude towards the social rights of immigrants than students of European origin. Almost all of them were from Melilla, which is in line with other studies conducted in this city [35,37]. This could be due to Melilla’s own cultural make-up [18] and the influence of this cultural context [38], which makes the Berber students share cultural and, in many cases, family ties with Morocco [39].

As for the academic year, first-year students exhibited the best attitudes towards immigrants, coinciding with the results obtained by Grueso and Arroyo [36], but at odds with the data obtained by Sánchez-Ojeda [35] and Keshet and Popper-Giveon [40]. Our data are worrying, since final-year students have been taught specific content on migration and cultural competence in the module Transcultural Nursing and have completed their hospital placements. They should therefore display better attitudes.

When analyzing the faculty variable, the results showed that students in Melilla and Ceuta exhibited the poorest attitudes, especially in relation to health and social care resources, which may be explained by the limited and scarce social care resources available, causing immigrants to be seen as competitors for these resources [12]. These data are consistent with studies conducted in Melilla, where nurses felt that the reason for their hospital being overwhelmed was that Moroccans were being treated there [41,42]. This perceived “abuse” of the healthcare services and social benefits by immigrants coincides with studies conducted in the general population [22,43]. However, this prejudiced perception has been proven to be untrue [23,33]. This perception is more related to the reduction in professionals and material resources that has been taking place in Spain since 2008 [44,45].

A number of studies have demonstrated that content on migration, cultural diversity, interculturality, and cultural competence taught in the nursing degree helps students to eliminate prejudicial and stereotyped attitudes towards immigrants and improves the care provided to immigrants [46,47,48,49]. Nevertheless, this content may be insufficient and further training may be needed to improve such attitudes not only among students, but also among nurses and other healthcare professionals [50,51,52], especially in a publicly funded universal healthcare system such as the Spanish one [33] where the negative attitudes of future nurses may translate into discriminatory behaviors [27] and violations of the recognized right of immigrants to accessing healthcare.

In response to the objective of this research, we may therefore assert that there are nursing students in Melilla, Ceuta, and Almeria who have negative attitudes towards the social rights of immigrants, and that greater intervention is needed to eliminate these attitudes, principally because they, as nurses, will have to care for patients from different cultures. As has been demonstrated in numerous researches, including cultural diversity and competence training improves the attitude of the students and minimizes negative beliefs and stereotypes towards cultural groups [10,14,15,53,54].

It is important to know nursing student’s attitudes towards immigrant people to be able to develop undergraduate measures for the acquisition of knowledge and cultural competence to offer the required tools to reduce negative attitudes and prejudices so that when these students become nursing professionals, such attitudes are decreased and they can offer competent care to the whole population regardless of the culture of the patients.

### Strengths and Limitations of this Study

The quantitative data of this study show the attitudes of nursing students towards the social rights of immigrants and the existing differences between the cultures, academic years, and faculties of the sample. The study has been developed in only three nursing faculties in the south of Spain so it would be interesting to expand it to other faculties with similar (and different) migratory pressure to be able to compare the results. The study methodology, an ex post facto design, offers a snapshot of these attitudes at a given time, which is limited by the data collection instrument used and does not reveal the causes of these attitudes. Further qualitative studies are needed to delve into these causes to be able to design interventions oriented to improve these attitudes. Although we have tried to avoid the influence of the research team in the interpretation of the results of the study, the researchers who conducted it are the professors of the universities under consideration. Consequently, errors, which have not been detected, could have existed.

## 5. Conclusions

These nursing students recognize the same social and labor rights for both the immigrant and native populations and uphold the right of undocumented immigrants to access publicly funded healthcare, which is a fact that should be taken into account as Spanish legislation recognizes that public healthcare is free and universal.

The students’ negative attitudes and their perceived abuse of health and social care resources by the migrant population, which are more pronounced among students in Melilla and Ceuta, reflect the prejudices that exist in society.

It is necessary to rethink and strengthen the intercultural training taught throughout undergraduate studies in order to ensure that existing prejudices disappear and that today’s students become culturally competent nurses tomorrow.

## Figures and Tables

**Table 1:** Percentages for the answers of the subscale “immigration and social rights”.

Positive Items	Faculty	SD (%)	D (%)	A (%)	SA (%)
We must make greater efforts to provide immigrants with a higher quality of life	Melilla	4.25	25.88	48.58	21.29
	Ceuta	4.29	23.62	50.64	21.45
	Almeria	4.29	21.02	48.03	26.66
It is desirable that immigrants be incorporated into our society as citizens with full rights	Melilla	2.12	14.90	58.86	24.12
	Ceuta	3.02	16.37	59.06	21.55
	Almeria	1.66	11.33	46.33	40.68
Immigrants should enjoy the same working conditions as nationals	Melilla	7.44	16.33	52.48	23.75
	Ceuta	6.46	19.39	49.13	25.02
	Almeria	2.34	10.36	45.48	41.82
I believe that undocumented immigrants should have full access to free education	Melilla	15.95	30.85	35.10	18.10
	Ceuta	11.68	32.04	36.37	19.91
	Almeria	14.06	25.75	32.44	27.75
All the people living in the same country, regardless of their origins or ethnicity, should have equal rights and obligations	Melilla	4.96	7.81	41.84	45.39
	Ceuta	1.29	8.62	45.26	44.83
	Almeria	1.33	5.01	27.65	66.01
**Negative items**	**Faculty**	**SD (%)**	**D (%)**	**A (%)**	**SA (%)**
Sometimes immigrants receive more social benefits than the locals themselves	Melilla	9.57	23.04	44.69	22.70
	Ceuta	8.93	20,44	41.27	29.36
	Almeria	20.66	26.02	34.66	18.66
Many immigrants take advantage of the health and social care resources that we have achieved through our efforts over many years	Melilla	11.72	25.17	42.19	20.92
	Ceuta	11.11	23.51	37.17	28.21
	Almeria	21.02	35.66	28.66	14.66
I believe that illegal immigrants and their families should NOT have access to publicly funded healthcare	Melilla	42.21	42.55	13.12	2.12
	Ceuta	32.75	46.98	13.79	6.48
	Almeria	51.01	38.33	6.33	4.33
To a great extent, publicly funded healthcare is overwhelmed by the increase in immigration	Melilla	15.63	32.26	37.58	14.53
	Ceuta	19.05	31.17	34.63	15.15
	Almeria	30.32	33.02	27.99	8.67
Too many resources are allocated to caring for immigrants	Melilla	17.37	50.72	26.95	4.96
	Ceuta	16.45	51.51	22.09	9.95
	Almeria	32.01	44.66	17.32	6.01
In the medium term, the massive arrival of immigrants will cause serious health and social problems	Melilla	7.81	34.04	42.55	15.60
	Ceuta	7.81	35.93	37.22	19.04
	Almeria	20.66	37.00	30.33	12.01
I believe that only the children of legal immigrants should have the right to go to school for free	Melilla	34.17	40.56	19.93	5.34
	Ceuta	27.51	42.80	23.14	6.55
	Almeria	44.34	33.67	13.33	8.66

SD: Strongly disagree; D: Disagree; A: Agree; SA: Strongly agree.

**Table 2:** Scores of the sum of positive and negative items on the social rights of immigrants.

Statistics		Subscale
***N***		821
***M***		25.971
**SD**		6.8064
**Min**		12.00
**Max**		46.00
**PC**	50	26.000
	75	31.000

*N*: Number of subjects for each type of prejudice; *M*: Mean of the scores obtained; SD: Standard Deviation; Min: Minimum score obtained; Max: Maximum score obtained; PC: Percentiles established for the selection of subjects.

**Table 3:** Immigration and Social Rights. Negative attitudes with statistically significant differences according to the variable: cultural background.

Immigration and Social Rights. Negative Attitudes	Mean Range European–Berber	Sig
Sometimes immigrants receive more social benefits than the locals themselves	408.71–277.75	0.000
Many immigrants take advantage of the health and social care resources that we have achieved through our efforts over many years	407.46–285.77	0.001
I believe that illegal immigrants and their families should NOT have access to publicly funded healthcare	404.22–310.17	0.000
In the medium term, the massive arrival of immigrants will cause serious health and social problems	402.91–324.77	0.007

*p* ≤ 0.05.

**Table 4:** Immigration and Social Rights. Positive attitudes according to the variable: academic year.

Items	Academic Year	*χ* ^2^	Mean Range	*p*
Immigrants should enjoy the same working conditions as nationals do	1st	15.515	365.52	0.001
	2nd		410.72	
	3rd		436.33	
	4th		433.65	
All the people living in the same country, regardless of their origins or ethnicity, should have equal rights and obligations	1st	16.191	365.74	0.001
	2nd		436.82	
	3rd		413.55	
	4th		430.53	

*χ*^2^: chi-squared test; *p* ≤ 0.05.

**Table 5:** Immigration and Social Rights. Positive and negative attitudes according to the variable: faculty.

Positive Items	Faculty	*χ* ^2^	Mean Range	*p*
It is desirable that immigrants be incorporated into our society as citizens with full rights	Melilla	24.546	428.00	0.000
	Ceuta		443.92	
	Almeria		360.07	
Immigrants should enjoy the same working conditions as nationals do	Melilla	33.910	440.44	0.000
	Ceuta		441.08	
	Almeria		349.02	
All the people living in the same country, regardless of their origins or ethnicity, should have equal rights and obligations	Melilla	32.431	441.57	0.000
	Ceuta		437.03	
	Almeria		352.64	
**Negative Items**	**Faculty**	***χ*^2^**	**Mean range**	***p***
Sometimes immigrants receive more social benefits than the locals themselves	Melilla	24.175	423.86	0.000
	Ceuta		453.05	
	Almeria		360.53	
Many immigrants take advantage of the health and social care resources that we have achieved through our efforts over many years	Melilla	34.014	432.15	0.000
	Ceuta		455.93	
	Almeria		349.28	
I believe that illegal immigrants and their families should NOT have access to publicly funded healthcare	Melilla	20.311	408.73	0.000
	Ceuta		454.86	
	Almeria		369.72	
To a great extent, publicly funded healthcare is overwhelmed by the increase in immigration	Melilla	23.650	441.80	0.000
	Ceuta		429.11	
	Almeria		357.27	
Too many resources are allocated to caring for immigrants	Melilla	19.603	428.76	0.000
	Ceuta		437.63	
	Almeria		362.96	
In the medium term, the massive arrival of immigrants will cause serious health and social problems	Melilla	24.426	435.74	0.000
	Ceuta		437.50	
	Almeria		356.50	
I believe that only the children of legal immigrants should have the right to go to school for free	Melilla	11.941	409.49	0.003
	Ceuta		441.09	
	Almeria		374.60	

*χ*^2^: chi-squared; *p* ≤ 0.05.
